# DNA Methylation Profile of Genes Involved in Inflammation and Autoimmunity in Inflammatory Bowel Disease

**DOI:** 10.1097/MD.0000000000000309

**Published:** 2014-12-02

**Authors:** Pantelis S. Karatzas, Gerassimos J. Mantzaris, Michael Safioleas, Maria Gazouli

**Affiliations:** From the 1^st^ Department of Gastroenterology, Evaggelismos Hospital, Athens, Greece (PSK, GJM); 4^th^ Department of Surgery, Attikon University Hospital, University of Athens, Athens, Greece (MS); and Department of Basic Medical Sciences, Laboratory of Biology, University of Athens, Athens, Greece (MG).

## Abstract

The contribution of epigenetic alterations to disease pathogenesis is emerging as a research priority. In this study, we aimed to seek DNA methylation changes in peripheral blood and tissue biopsies from patients with inflammatory bowel disease.

The promoter methylation status of genes involved in inflammation and autoimmunity was profiled using the Human Inflammatory Response and Autoimmunity EpiTect Methyl II Signature PCR Array profiles. Methylation was considered to be hypermethylated if >20% according to the instructions of the manufacturer. The microarrays were validated with Quantitative Real-time PCR.

Regarding Crohn disease (CD) no gene appeared hypermethylated compared to healthy controls. In ulcerative colitis (UC) 5 genes (*CXCL14*, *CXCL5*, *GATA3*, *IL17C*, and *IL4R*) were hypermethylated compared to healthy controls. Some of the examined genes show different methylation patterns between CD and UC. Concerning tissue samples we found that all hypermethylated genes appear the same methylation pattern and confirmed a moderate–strong correlation between methylation levels in colon biopsies and peripheral blood (Pearson coefficients *r* = 0.089–0.779, and *r* = 0.023–0.353, respectively).

The epigenetic changes observed in this study indicate that CD and UC exhibit specific DNA methylation signatures with potential clinical applications in IBD non-invasive diagnosis and prognosis.

## INTRODUCTION

Inflammatory bowel diseases (IBD), encompassing ulcerative colitis (UC) and Crohn disease (CD), are life-long, idiopathic, polygenic diseases with significant genetic heterogeneity. Despite significant advances in understanding the mechanisms that participate in the development of IBD, the exact pathogenesis remains mysterious.^1^ Current literature supports that IBD is the result of an aberrant immune response against commensal bacteria and luminal antigens in a genetically susceptible host.^2^ Genetic studies, including candidate gene approaches, linkage mapping studies, and genome-wide association studies (GWAS), have significantly advanced our understanding on the importance of genetic susceptibility in IBD.^3,4^ The meta-analysis of several GWAS performed so far has identified more than 30 risk-conferring loci. Another important information emerged from GWAS is that defects in autophagy are also associated with susceptibility to CD and that Th17 pathways are involved both in CD and UC.^4,5^ However, by associating genotypes with clinical outcome little can be inferred on the disease-causing mechanisms. This suggests that additional factors—including epigenetic factors such as DNA methylation—are involved in the pathogenesis of IBD. Toyota et al^6^ were the first to show that high grade dysplasia in inflamed colonic mucosa was associated with hypermethylation of specific genes, suggesting that chronic inflammation is associated with increased levels of methylation. Abnormal DNA methylation has also been observed in UC patients in the estrogen receptor (*ER*), *P14ARF*, and *E-cadherin* gene.^7^ Recently, Cooke et al^8^ reported several genes which showed significant evidence of differential methylation both in UC and CD and many more than expected by chance overlapped with genes that were previously implicated in IBD susceptibility in GWAS. Since inflammation and autoimmunity are the basic mechanisms in the pathogenesis of IBD, the aim of this study was to evaluate the impact of differences in methylation patterns in the peripheral blood and tissue biopsies using a platform-based array technology with regard to IBD susceptibility. Additionally, as blood is one of the most easily available (non-invasive) clinical samples and “pathologic” DNA is known to be present in blood stream^9^ we intended to define whether differences in methylation of tissue samples are in concordance to blood samples.

## MATERIALS AND METHODS

### Patient Samples

Genomic DNA was isolated from whole peripheral blood and intestinal biopsies of 24 adult patients with newly diagnosed active IBD (12 CD, 4 male, and 8 female; and 12 UC, 8 male, and 4 female), before the initiation of any kind of treatment. The diagnosis of either CD or UC was based on standard clinical, endoscopic and histological criteria. UC cases were phenotyped according to the Montreal Classification system; 8 patients had pancolitis, and 4 left sided colitis. Concerning CD, 2 patients had colitis, 4 ileocolitis and 6 ileitis, whereas the disease behavior was inflammatory in all 12 patients. 12 whole blood DNA samples from healthy individuals with no evidence of gastrointestinal disease or family history of IBD served as controls. Patients and controls were non-smokers. The mean age of the CD patients was 31.92 ± 8.51 years, of the UC patients’ 32.13 ± 6.12 years, and of the controls’ 41.81 ± 10.51 years. The DNA was isolated using the Nucleospin Tissue kit (Macherey-Nigel GmbH & Co, Düren, Germany). The study was approved by the Ethics Committee at Evangelismos Hospital and all study subjects provided informed consent.

### Methylation PCR Analysis

The restriction digestions were performed using the EpiTect Methyl II DNA Restriction Kit provided by Qiagen (Chatsworth, CA) according to the manufacturer's instructions and as previously described in other studies.^10^ A reaction mix without enzymes was prepared from 1 μg genomic DNA, 26 μL of 5× Restriction Digestion Buffer, and RNase-DNase free water to make the final volume 120 μL. Four digestion reactions (Mo, Ms, Md, and Msd) were set up. Each one consists of 28 μL of the previous reaction mix and 2 μL of RNase-DNase free water for Mo digest; 28 μL of the previous reaction mix, 1 μL of methylation sensitive enzyme A and 1 μL of RNase-DNase free water for Ms digest; 28 μL of the previous reaction mix, 1 μL methylation sensitive enzyme B and 1 μL of RNase-DNase free water for Md digest; 28 μL of the previous reaction mix, 1 μL of methylation sensitive enzyme A and 1 μL of methylation sensitive enzyme B to make the final volume 30 μL for Msd digest. All 4 tubes were incubated at 37°C for O/N. The digested DNA samples were then analyzed using the Human Inflammatory Response and Autoimmunity EpiTect Methyl II Signature PCR Array Profiles (Qiagen, Chatsworth, CA) according to the manufacturer's instructions and as previously described.^10^ The method is based on the detection of remaining input DNA after cleavage with methylation-sensitive and methylation-dependent restriction enzymes. The Human Inflammatory Response & Autoimmunity EpiTect Methyl II Signature PCR Array profiles the promoter methylation status of a panel of 22 genes [Chemokines: *CCL25*, *CXCL14*, *CXCL3*, *CXCL5*, *CXCL6*, Cytokines: *IL12A*, *IL12B*, *IL17C*, *IN*HA, Cytokine Receptors and Associated Proteins: *IL10RA*, *IL13RA1*, *IL15*, *IL17RA*, *IL4R*, *IL6R*, *I*L6ST, Other Inflammatory Response & Autoimmunity Genes: *ATF2*, *FADD*, *GATA3*, *IL13*, *IL7*, *TYK2*]. The Real-time PCR assay was performed in an ABI PRISM 7000 Sequence Detection System (Applied Biosystems, Foster City, CA). The data were analyzed using the EpiTect Methyl II PCR Array System. The system provides an integrated Excel-based template that automatically performs all ΔCt based calculations from the raw threshold cycle (Ct) values to determine gene specific DNA methylation status, using MethylScreen™ technology. The Excel template normalizes the Ct values of both digests with the mock digestion values to calculate and report the percentage of the DNA that is methylated and unmethylated. The minimum level of hypermethylation considered to be positive is set at 20%. All experiments were performed in duplicates.

### Quantitative Real-Time PCR (qRT-PCR)

Expression of selected methylated genes (*CCL25*, *IL13, IL12B*, *CXCL5*, *IL4R*, *IL17RA*, and *IL17A*) was analyzed by qRT-PCR using the Applied Biosystems ABI PRISM 7000 Sequence Detection System. RNA was extracted from whole blood using a DNA/RNA All Prep minikit (Qiagen) and RNA was converted to cDNA using a high-capacity reverse transcription kit (Applied Biosystems) according to the manufacturer's instructions. Samples were analyzed in duplicates, and genes of interest were compared to GAPDH as endogenous reference. Relative quantification of gene expression was calculated using the ΔΔCT method. Analysis of relative gene expression data was made using real-time quantitative PCR and the 2(−Delta Delta C(T)) method.

### Statistical Analyses

Statistical analysis was performed using Statistical Package for Social Sciences, Version 20.0 (SPSS Inc, Chicago) for Windows. Statistical analysis of the EpiTect Methyl II Signature PCR Array is described above.

Methylation was considered to be hypermethylated if >20% according to the instructions of the manufacturer. Kruskal–Wallis analysis was used to detect the existence of any possible statistical significant difference among the three groups. The level of statistical significance was set after the Bonferroni correction at *P* = 0.0166. Differences in methylation levels were evaluated using the non-parametric Mann–Whitney test. Pearson correlation coefficient was calculated between blood and tissue samples.

## RESULTS

Clustering results on paired differences of our whole blood sample set showed significant methylation changes between active CD, active UC and normal samples for the CpGs analyzed in the Human Inflammatory Response and Autoimmunity EpiTect Methyl II Signature PCR Array. The differential genes methylation for each of the above comparisons is shown in Table 1.

**TABLE 1:**
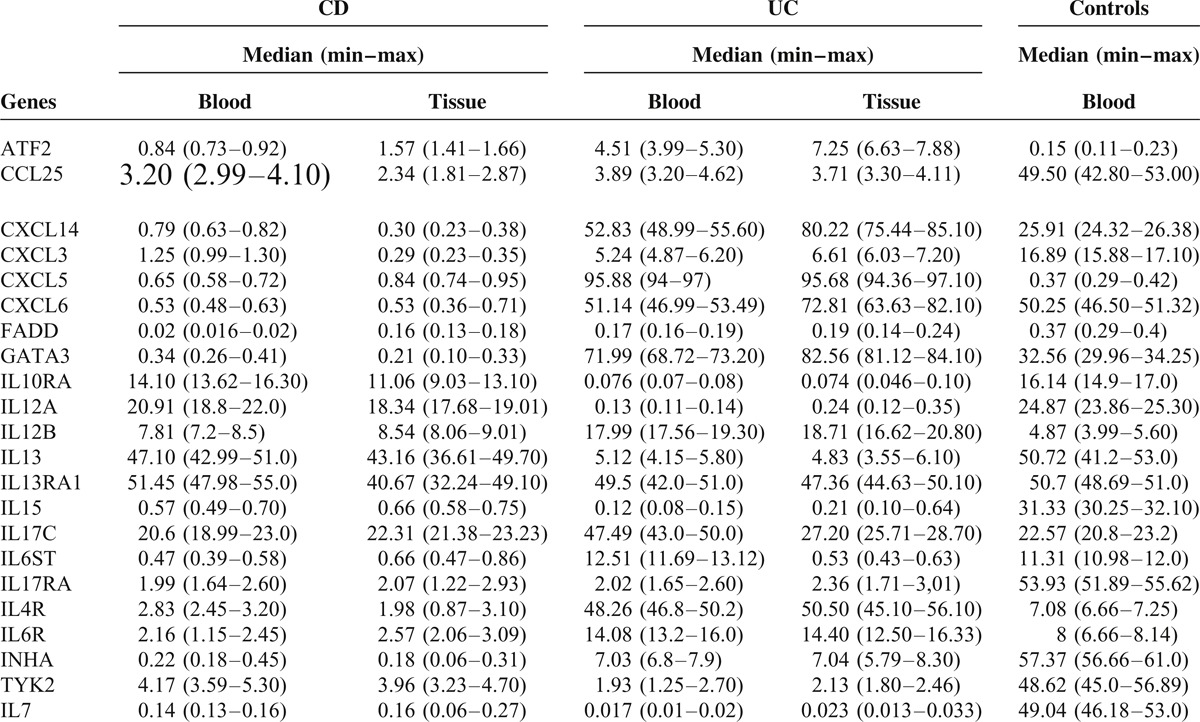
Gene Methylation Status Presented as the Median of the Percentage Methylated (M) Fraction of Input Genomic DNA Containing Two or More Methylated CpG Sites in the Targeted Region of a Gene

Regarding CD, the methylation status of *IL10RA*, *IL13*, *IL13RA1*, and *IL17C* in peripheral blood samples did not differ significantly from the methylation status of healthy controls. Only three genes—*ATF2*, *CXCL5*, and *IL12B* showed higher methylation in CD compared to controls, but they did not exceed the threshold of 20% for hypermethylation. All other genes tested appear lower methylation than controls (Table 1).

Regarding UC, methylation status of *CXCL6* and *IL13RA1* in peripheral blood samples did not differ significantly from the methylation status of healthy individuals. Five genes (*CXCL14*, *CXCL5*, *GATA3*, *IL17C*, and *IL4R*) were found to be significantly hypermethylated in UC patients compared to healthy individuals (*P* = 0.002). Especially *CXCL5* and *IL4R* appear great difference in methylation pattern between UC and controls, as these two genes are highly methylated in UC and extremely low methylated in controls (95, 88 vs 0, 37 and 48, 26 vs 7, 08 respectively). Some genes show higher methylation than controls, but they do not exceed 20% methylation threshold for hypermethylation (Table 1). All other genes show lower methylation in UC compared to controls.

Active cases of both CD and UC could be promptly distinguished from healthy controls based on the signatures provided by the methylation profiles in peripheral blood samples. Based on these results—despite the relatively limited number of patients—it was possible to define distinct signatures for active CD vs active UC (Table 2). Specifically, *CXCL14*, *CXCL5*, *GATA3*, *IL17C*, and *IL4R* genes were hypermethylated in UC compared to CD (*P* = 0.002). In addition, *CXCL6* which did not differ significantly between UC and controls, appeared hypermethylated compared to CD; in contrast, *IL13* which did not differ significantly between CD and controls appeared hypermethylated in CD compared to UC (Table 1).

**TABLE 2:**
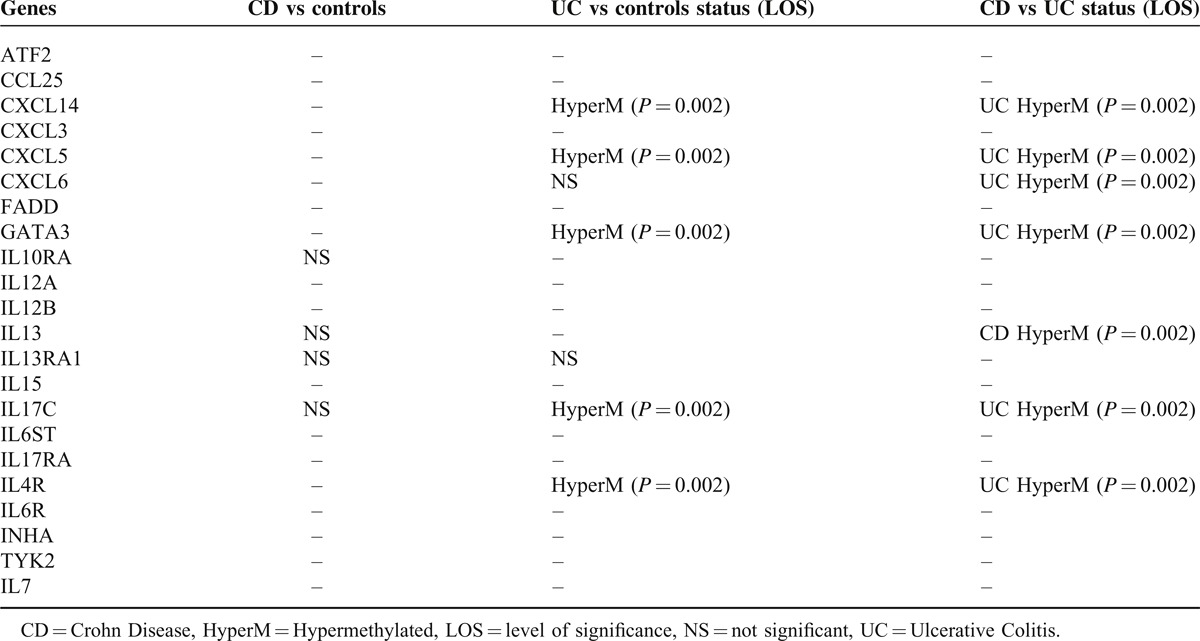
Genes Showing Statistically Significant Evidence of Hypermethylation and Differential Methylation Among Active CD, UC, and Controls

**FIGURE 1:**
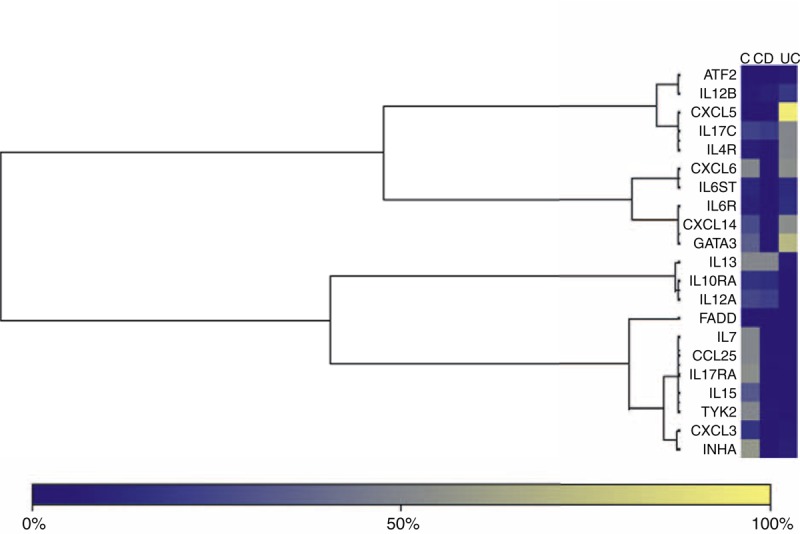
Representative Heatmap of the genes and samples for each comparison. Microarray heatmaps use a colored grid linked by a dendrogram of the samples to hierarchically cluster genes. Each cell in the heatmap is colored based on the level of expression of that probe in that sample. Yellow cells signify an increase and blue a decrease in methylation. UC, active ulcerative colitis; CD, active Crohn disease; control, healthy samples. C = controls, CD = Crohn disease, UC = Ulcerative colitis

According to the literature epigenetic alterations have been repeatedly confirmed in colonic mucosa, whereas limited knowledge exists regarding peripheral blood specimens. Therefore we have tested respective colon biopsies from these 24 IBD-patients in order to examine if methylation profile is in concordance to that of blood specimens. All tested genes that appear different methylation profile among CD, UC and controls show moderate–strong correlation between methylation levels in colon biopsies and methylation levels in peripheral blood both in CD and UC patients (Pearson coefficients *r* = 0.089–0.779, and *r* = 0.023–0.353, respectively). The differential genes methylation for each of the above comparisons is shown in Table 1 and Figure 1.

**FIGURE 2:**
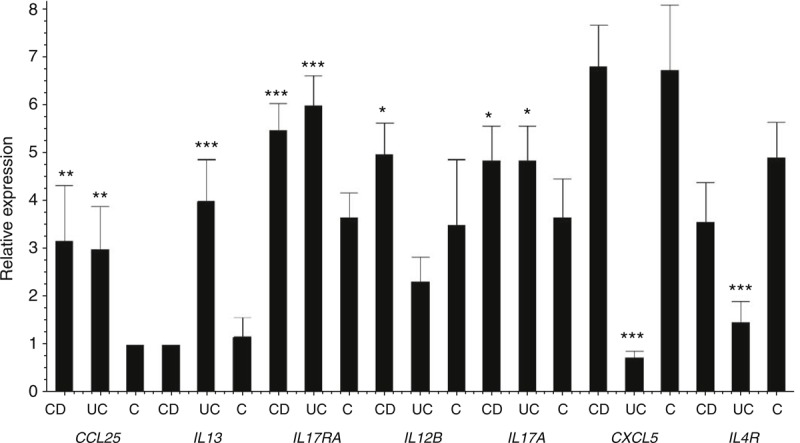
Quantitative RT-PCR data. Comparison of mRNA expression levels between CD, UC, and control samples.

Having obtained evidence for the presence of differential methylation between CD cases, UC cases and controls, important questions raise regarding the functional impact of such signals on gene transcription. The former was addressed by undertaking qRT-PCR for selected gene targets that have shown evidence of marked differential methylation on the array experiment. *CCL25*, *IL13*, *IL12B*, *CXCL5*, *IL4R*, and *IL17RA* were selected from the aforementioned genes and studied with relative quantification data revealing changes between cases and controls. Moreover we performed qRT-PCR for *IL17A*, a gene known to play central role as pro-inflammatory cytokine in the pathogenesis of IBD.^11^ Increased expression of mRNA levels was observed in cases with reduced methylation as well as for *IL17A* (Figure 2).

## DISCUSSION

Recent evidence suggests that pathways involved in the pathogenesis of IBD include those of pro-inflammatory cytokines, pattern recognition receptor signals, cytokines, growth factors, heat-shock proteins, epithelial cyto-/chemokines regulating migration, activation and Ig class switching, autophagy and many others. Some of the known susceptible loci, implicated in many different molecular pathways, are common in UC and CD, whereas others are specific for each disease. Autophagy genes (eg, *ATG16L1*, *IRGM*), NOD-like receptors (eg, *NOD2*) and intelectins (*ITLN1*) are highly specific for CD, whereas loci related to regulatory pathways (*IL10* and *ARPC2*), intestinal epithelial cell (IEC) function (eg, *ECM1*) and E3 ubiquitin ligase (eg, *HERC2*) appear to be specific for UC.^2,3,12–14^ According to the existing literature, a large number of studies has shown the increased expression of the molecules implicated in the aforementioned pathways but only few have documented methylation as a causative factor. In this respect, epigenetic mechanisms—and more specifically DNA methylation—seem to be of great importance,^15^ but the number of the existing studies on methylation in peripheral blood of IBD patients remains limited. Based on the belief that methylation levels in peripheral blood may be used as a minimally invasive biomarker, we conducted a study on methylation level of genes implicated in the most common pathways of IBD, collected from peripheral blood and intestinal tissue. The need for a reliable biomarker is profound as none of the available biomarkers is sufficiently specific for the diagnosis of IBD or the follow up of patients. Serum C-Reacting Protein (CRP) and less so fecal calprotectin are widely used biomarkers in assessing the activity of IBD and following the course of the disease, but both lack specificity whereas their sensitivity is questionable in limited disease.^16^ Methylation levels, as shown already in previous studies, may be more closely and specifically related to disease activity and thus play a role as a more reliable biomarker.^17–20^

Genes implicated in some of the most well-known pathways of IBD pathogenesis, such as chemokines, cytokines, inflammatory response and autoimmunity genes have been studied. It must be noted that the majority of the genes selected have not been studied for methylation until now. Three different groups were created: controls, CD patients and UC patients. We tested for methylation levels in blood samples as well as intestinal tissue samples (for CD and UC patients) in order to estimate if methylation profile in tissue and blood are in concordance. CD and UC patients had active disease. Using strict statistical analysis, we compared the results of each gene among all groups; that is between controls and CD or UC patients as well as between UC and CD patients. Out of the 22 genes studied, only *IL13RA* showed no statistically significant differences among the three groups in blood and tissue samples. No significant differences were found in methylation of *IL10RA*, *IL13*, and *IL17C* between controls and CD patients. *CXCL6* methylation did not differ significantly between controls and UC patients.

Regarding CD, three genes (*ATF2*, *CXCL5*, and *IL12B*) show—statistically—higher methylation than controls but they are not hypermethylated according to the 20% threshold set for the specific experiment.

Regarding UC, five genes (*CXCL14*, *CXCL5*, *GATA3*, *IL17C*, and *IL4R*) appeared hypermethylated in UC patients compared to controls, indicating a potential role in the pathogenesis of UC. *CXCL14* is a cytokine expressed at high levels in normal tissues but reduced or absent in cancer cells. Its role is chemotactic for monocytes in the presence of prostaglandin-E2. Recently hypermethylation of its’ promoter region was related to lower expression of *CXCL14* in cases of gastric cancer.^21^*CXCL5* is a cytokine that stimulates the chemotaxis of neutrophils but also regulates neutrophils homeostasis. It originates mainly from enterocytes and is elevated in human colonic tissues from patients with UC.^22^

The role of interleukins (IL) in UC is well established and therefore hypermethylation of *IL17C* and *IL4R* in our study was of no surprise.^23–25^ Apart from identifying genes showing different methylation levels between IBD cases and controls, we also conducted a qRT-PCR in order to demonstrate the functional impact of altered methylation on expression of selected genes. Lower methylation of the selected genes (*CCL25*, *IL13*, *IL12B*, *CXCL5*, *IL4R*, *IL17A*, and *IL17RA*) was related to higher levels of mRNA expression. Specifically *IL13* confirms this relationship as the higher methylation levels in CD compared to UC is related to lower levels of mRNA in CD compared to UC. Moreover qRT-PCR of *IL17A* confirmed higher expression in CD and UC compared to controls in concordance to the methylation and mRNA expression pattern of *IL17RA*. Systematic testing of all other genes showing differential methylation was beyond the scope of the current study, but we have no reason to suppose that these would be different.

Another interesting observation was the different level of methylation of specific genes between CD and UC. Especially *CXCL14*, *CXCL5*, *CXCL6*, *GATA3*, *IL17C*, and *IL4R* appeared hypermethylated in UC compared to CD, whereas *IL13* was hypermethylated in CD compared to UC. Although UC and CD share common pathways, they also exhibit unique pathogenetic features. Therefore it seems reasonable that some genes show diametrically different levels of methylation, but the fact that these differences can be detected in peripheral blood specimens poses a new perspective in the use of methylation as a biomarker and especially as a tool to differentiate UC from CD in some dubious clinical cases. In other words, the different methylation levels between CD and UC could play a role in confirming the diagnosis of IBD, determining the exact type of IBD (UC or CD) and possibly estimating the activity of disease. The fact that hypermethylation of these genes in peripheral blood and intestinal tissue biopsies are in concordance enhances the assumption that peripheral blood methylation could be used as a biomarker.

There are reports that raise concerns about the use of whole blood DNA on methylation studies due to the heterogeneity of different cell types, each having their own particular epigenome, which can confound DNA methylation measurements.^26,27^ Nonetheless, we observed that methylation patterns of intestinal tissue and peripheral blood are in concordance. Additionally some of the hypermethylated genes show extreme differences in methylation (eg, *CXCL5* with 95.88% methylation in UC, 0.65% in CD and 0.37% methylation in controls), which cannot be explained by confounding factors only.

In conclusion, in this study we have identified panels of genes that show evidence of differential methylation between CD and controls, UC and controls, and CD and UC. Moreover there is strong evidence that methylation level in intestinal tissue samples is well related to methylation level in whole blood samples. Our findings suggest that these genes play an important role in IBD pathogenesis, and gene set analysis has highlighted a number of key pathways. The differential methylation status observed affects gene expression at the mRNA level. However larger studies on IBD-associated changes in DNA methylation are required and may lead to clinical application in disease diagnosis and prediction of phenotype outcome.
